# Help-seeking and antibiotic prescribing for acute cough in a Chinese primary care population: a prospective multicentre observational study

**DOI:** 10.1038/npjpcrm.2015.80

**Published:** 2016-01-21

**Authors:** Carmen Ka Man Wong, Zhaomin Liu, Chris C Butler, Samuel Yeung Shan Wong, Alice Fung, Dicken Chan, Benjamin Hon Kei Yip, Kenny Kung

**Affiliations:** 1 Division of Family Medicine and Primary Health Care, JC School of Public Health and Primary Care, Faculty of Medicine, The Chinese University of Hong Kong, Hong Kong, China; 2 Nuffield Department of Primary Care Health Sciences Oxford University, New Radcliffe House, Radcliffe Observatory Quarter, Oxford, UK; 3 Cardiff University, Institute of Primary Care and Public Health, Cardiff, Wales; 4 Department of Family Medicine and Primary Care, University of Hong Kong, Hong Kong, China

## Abstract

Acute cough is a common reason to prescribe antibiotics in primary care. This study aimed to explore help-seeking and antibiotic prescribing for acute cough in Chinese primary care population. This is a prospective multicentre observational study that included adults presenting with acute cough. Clinicians recorded patients’ presenting symptoms, examination findings and medication prescription. Patients completed symptom diaries for up to 28 days by charting their symptom severity and recovery. Adjusted binary logistic regression models identified factors independently associated with antibiotic prescription. Primary care clinicians (*n*=19) recruited 455 patients. A total of 321 patients (70.5%) returned their completed symptom diaries. Concern about illness severity (41.6%) and obtaining a prescription for symptomatic medications (45.9%), rather than obtaining a prescription for antibiotics, were the main reasons for consulting. Antibiotics were prescribed for 6.8% (n=31) of patients, of which amoxicillin was the most common antimicrobial prescribed (61.3%), as it was associated with clinicians’ perception of benefit from antibiotic treatment (odds ratio (OR): 25.9, 95% confidence interval (CI): 6.7–101.1), patients’ expectation for antibiotics (OR: 5.1, 95% CI: 1.7–11.6), anticipation (OR: 5.1, 95% CI: 1.6–15.0) and request for antibiotics (OR 15.7, 95% CI: 5.0–49.4), as well as the severity of respiratory symptoms (cough, sputum, short of breath and wheeze OR: 2.7–3.7, all P<0.05). There was a significant difference in antibiotic prescription rates between private primary care clinicians and public primary care clinicians (17.4 vs 1.6%, P=0.00). Symptomatic medication was prescribed in 98.0% of patients. Mean recovery was 9 days for cough and 10 days for all symptoms, which was not significantly associated with antibiotic treatment. Although overall antibiotic-prescribing rates were low, there was a higher rate of antibiotic prescribing among private primary care clinicians, which warrants further exploration and scope for education and intervention.

## Introduction

Antibiotic prescription for respiratory tract infection accounts for a major proportion of antibiotics used in primary care, and it contributes to the rising prevalence of resistance among major human pathogens. Acute cough is a common presentation to primary care, and antibiotic prescription rates can range from 20 to 90% in European countries.^1–3^ It has been shown that antibiotics have marginal effects for otherwise healthy individuals,^4–6^ and antibiotic prescribing has little impact on clinical recovery.^1^ Interventions have been developed to promote more appropriate help-seeking and antibiotic prescribing that take into account patient variation in perceptions and expectations for antibiotic prescription.^7^ Illness severity, country of residence, age, attitude to the doctor and attitude to antibiotics have all been associated with differences in antibiotic prescriptions.^8,9^ However, there is a lack of evidence regarding antibiotic prescribing for respiratory tract infections in Asia, despite high levels of antibiotic resistance. Understanding the pattern of presentation, the influence of patient perceptions and expectations and threshold for antibiotic prescription is important for informing antimicrobial stewardship interventions for primary care physicians. Therefore, this study aimed to explore (1) symptoms and duration of acute cough, (2) patient perception and expectations for antibiotics and (3) antibiotic prescription and associated factors. We hoped to inform interventions aimed at enhancing appropriate antibiotic use in primary care in Asia.

## Results

In total, 455 patients were recruited from 19 participating clinicians, of which 67.3% of patients were recruited from public primary care clinics and 32.7% of patients from private clinics. The recruitment period was from November 2011 to February 2014. Case report forms were returned for all patients, and diaries were returned from 321 (70.5%) patients. All case reports and patient diaries received were included in item analysis, as missing data did not exceed 5% for each questionnaire. The mean age of patients was 47.1±14.9 years and 43% (138/321) were men (Table 1). The mean time for patients’ overall symptom recovery was 9.9±7.0 days. Clinicians’ assessment, management and perception on antibiotics prescription is indicated in Table 2. The total symptom severity score at first consultation rated by clinicians was 11.1±5.5. In all, 18.5% of patients had oral temperature ⩾37.2 °C. Antibiotics were prescribed for 6.8% (*n*=31) of included patients, of which amoxicillin accounted for 61.3% (*n*=19) of prescriptions. Of those who did not fill in a diary (*n*=136), 5.1% (7/136) of patients were prescribed antibiotics.

Regarding clinicians’ perceptions of patient views, 10.2% (*n*=46) agreed or strongly agreed that the patient wanted them to prescribe antibiotics, among whom 94.0% (*n*=428) perceived the patient to be satisfied with their consultation and 8.6% (*n*=39) of clinicians believed that antibiotics can help quick recovery.

Table 3 indicates patients’ perceptions on antibiotic prescription and their satisfaction with the consultation. Most of the patients (85%) were satisfied with the consultation. Overall, 14.3% (*n*=45) of patients reported that they expected an antibiotic prescription, 10.2% (*n*=32) reported that they were hopeful for antibiotics and 2.9% (*n*=9) reported that they requested antibiotics in the consultation. In all, 65.4% of patients believed that antibiotic use will increase resistance. Concern about illness severity (41.6%) and obtaining a prescription for symptomatic medications (45.9%), rather than obtaining a prescription for antibiotics, were the main reasons for consulting.

Binary logistic regression models (Table 4) indicate that after adjustment for potential covariates the strongest predictors of antibiotic prescribing included the following: clinicians’ perception on the benefit of antibiotics (odds ratio (OR): 25.9, 95% confidence interval (CI): 6.7–101.1), patients’ expectation (OR: 5.1, 95% CI: 1.7–11.6), anticipation (OR: 5.1, 95% CI: 1.7–15.0) and request (OR 15.7, 95% CI: 5.0–49.4) for antibiotics. The severity of respiratory symptoms such as cough, sputum, short of breath and wheeze (OR 2.7–3.7, *P*<0.05 (all)) were also associated with increased antibiotic prescribing. Patients’ belief that antibiotic use will increase resistance was associated with reduced likelihood of prescribing (OR: 0.494, 95% CI: 0.307–0.794). The total symptom scores rated by clinicians were marginally associated with antibiotic prescribing (OR: 1.113, *P*=0.022). Private primary care clinicians were more likely to prescribe antibiotics (OR:9.7, 95% CI: 2.0–46.0).

There were no statistically significant associations between antibiotic use and patients’ symptom recovery (overall, cough/sputum, cough and sputum: *P*>0.05 (all); Table 5). However, prescription of antibiotics was associated with better patients’ satisfaction (OR: 2.8, 95% CI: 1.3–3.9).

An autoregressive moving average (1,1) model comprised first-order autoregressive part and first-order moving average part. Both the main effect of antibiotic prescription and its interaction with time were included in the model, with adjustment of age, gender, season, years of education, smoking status, duration of illness before consultation and coexisting medical conditions. This allowed comparison of recovery rates over time for those with and without antibiotics. Interaction term between prescribing antibiotics and day of presentation in autoregressive moving average model was not significant (parameter estimate=−0.079, *P* value=0.353); therefore, being prescribed antibiotics was not associated with a faster reduction in symptom severity scores.

Of the primary care consultations, 17.4% in private care settings resulted in antibiotic prescription (*n*=149) compared with only 1.6% of consultations in a public care setting (*n*=306), which was statistically significant (*P*=0.00). Patient profiles presenting to public healthcare settings show a difference in the comorbidity profile such as diagnosed ischaemic heart disease, heart failure and diabetes compared with those presenting to private health care (*P*<0.05 (all)). However, there were no significant differences in comorbidity profile in terms of chronic obstructive pulmonary disease and other lung disease, although asthma was more prevalent in private settings (12.7% vs 6.5%, *P*=0.03).

Antibiotic prescribing in patients with chronic obstructive pulmonary disease, asthma or other lung disease only constituted 25.8% (*n*=8) of all antibiotic prescriptions, although antibiotics were statistically significantly prescribed by private primary care clinicians (Supplementary Table 1). Private primary care clinicians were also more likely to perceive illness as severe, although patient ratings of illness severity were similar and the association of clinician-rated severity with antibiotic prescribing was marginal (Table 4 and Supplementary Table 2). Almost all patients (98%) were given symptomatic medication. Of those, the commonest prescribed medication included cocillana syrup, mistura expectorants stimulants and bisolvon (bromohexine), as well as paracetamol and NSAIDs.

## Discussion

### Main findings

Physicians’ prescription of antibiotics for acute cough was low (6.8%) in this prospective study of presentation and management of acute cough in Hong Kong primary care. Symptom recovery took a median of 10 days, and clinical recovery of acute cough was not associated with antibiotic prescription. Use of symptomatic medication was almost universal, but there were significant differences in antibiotic prescription between private and public primary care clinicians (17.4 vs 1.6%).

### Interpretation of findings in relation to previously published work

In this study, the rate of antibiotic prescription for acute cough was much lower than in other European countries^1^ and Mainland China.^8,10^ Antibiotic prescription for acute cough ranged from 21 to 75% in Europe.^1^ In mainland China, antibiotics were prescribed for 78.0% of respiratory tract infections and 93.5% of cases of acute bronchitis^10^ and almost universally in treating diarrhoea and cough.^8^ Amoxicillin was most commonly prescribed, which was similar to UK data.^1^ Patient profiles of participants in terms of an existing respiratory condition (chronic obstructive pulmonary disease, asthma or other condition) were similar with European counterparts^1^ (15.4% vs 15.3%), although the rate of smoking was much lower in our study (6.3% vs 28%). In addition, patients sought medical care earlier, as patients were unwell for a median of 2.0 days (quartile range 2–4 days) in comparison with 5 days in Europe. The mean symptom severity score was 11.1, which was lower than other European countries. Patient expectations for antibiotics was lower than in Europe in similar study settings ranging from 25.4 to 73.2% (mean 45.1%) compared with our study findings of 14.3%.^11^ Low symptom severity, short duration of illness before presentation and low patient expectations for antibiotics in our study may partly contribute to physicians’ scarce use of antibiotics.

Influences on antibiotic prescription can include clinician perception of medical need,^7^ patient satisfaction^12^ and in protecting the patient-clinician relationships by not prescribing antibiotics.^13,14^ In our study, the influence of clinicians’ and patients’ perceptions for antibiotics significantly influenced the decision of antibiotic prescribing, and this appears consistent with international observations.^7,12,15^

Interestingly, prescription of symptomatic relief medications was almost universal, in contrast to European countries where less than half of patients are prescribed symptomatic medications.^16^ Symptom recovery took a median of 9.9 days in comparison with 11.0 days in the European study. Differences in overall symptom duration inclusive of days before presentation (12 vs 16 days^1^) may be attributed to differences in presenting symptoms/ illness or help-seeking behaviour or may suggest a role of symptomatic medication in symptom recovery. In Hong Kong, the primary care providers include public primary care physicians, private primary care physicians and traditional chinese medicine doctors. Private primary care physicians see the majority of primary care consultations, and fees are 200HKD or above (1 USD=7.8 HKD). Fees in the public sector are subsidised, and patients pay 45HKD. The cost of symptomatic medication of 3–4 days and/or 1-week course of antibiotics are usually included in the consultation cost in both sectors. Cultural or institutional factors intrinsic to the primary care system in Hong Kong, e.g., patient perceptions of illness, use of Chinese traditional medicine, healthcare system and access, may also contribute to the variations in medical treatment, which may explain why some patients expected or hoped for antibiotics but did not view this as their main reason for consultation above their concern about illness severity and in receiving symptomatic treatment. The antibiotic prescription rates in our study are much lower than documented rates in China;^10^ this may partly be because of differences in health system setup, school and medical education of clinicians, as well as public education influenced under British rule. This can be further explored by comparative cross-cultural qualitative studies of patients and doctors.

Our finding that antibiotic use was not associated with improved recovery supports the increasing evidence that antibiotics do not benefit most adult patients with acute cough/lower respiratory tract infection who are otherwise well.^1,17,18^ Our study findings that associate antibiotic prescribing with clinicians’ perception of illness severity, benefit, patients’ expectation, anticipation and requests, suggests the influence of these factors on the clinician’s decision may be inherent to the setting (e.g., public or private). Further exploration on the role of fees, clinic access, patient expectation and doctors’ perceptions is warranted to tailor doctor and patient education and effective interventions across different primary care settings.

### Strengths and limitations of this study

This is the first prospective multicentre observational study of acute cough and antibiotic prescription in Asia. Understanding possible differences, cultural influences and similarities of patients’ perceptions and clinicians’ use of antibiotics is important in addressing inappropriate antibiotic prescribing. The consistent association of patients’ and clinicians’ perceptions on antibiotic prescription appears to be universal. The observational nature of this study can enhance the congruency of our findings to current practice. However, limitations of this study design include the inability to account for unknown confounders. In addition, patient recovery was self-assessed by patients. However, studies have suggested that self-report by diary has been shown to have moderate-to-high concordance with measurement of adherence through objective measures.^19^ Clinicians may also adhere more to best practice recommendations when they are part of a research study, resulting in an underestimation of antibiotic prescriptions. The overall response rate to the diary was slightly less than the original GRACE study. However, the number of returned and completed patient diaries exceeded all but one of the participating countries,^1^ whereas the number of participating clinicians and clinic recruitment sites were comparable. The duration of recruitment took longer than expected with staggered recruitment periods of the clinics to reach target recruitment. Because of the lack of data collection of those not recruited, there is a risk of recruitment bias, in which clinicians may recruit those in whom they were less likely to prescribe antibiotics. Limitations of the GRACE study extend to our study in which the participating clinicians and patients may not have been representative of the whole region/country. Our study patients are representative in terms of age, education and occupation status, household size and smoking status of the general Hong Kong population.^20,21^ In addition, the relatively broad inclusion criteria in our study captured a wide range of patients with cough/LRTI, which may increase the generalisability of our results, while the possible selection and response bias might have underestimated the associations of factors with antibiotics prescription. Studies in the region for upper respiratory tract infections report a decline of antibiotic prescription from 8.1 to 5.1% from 2005 to 2010 in the public primary care sector,^22^ which is consistent with our study finding of 6.8% in acute cough in a mixed private and public primary healthcare setting.

### Implications for future research, policy and practice

Our findings show influences on antibiotic prescribing consistent with clinicians’ perception of benefit, patients’ expectation, anticipation and requests in addition to presenting symptoms. A higher proportion of antibiotic prescribing by private primary care clinicians may also be evident in other health systems particularly in the Asia Pacific region where there is a mixed public and private primary care provider system. Patient and clinician factors can be further explored in relation to public and private primary care settings to develop effective interventions and tools in helping clinicians resist overbearing patient demands for appropriate use of antibiotics. The overall low usage of antibiotics may be placated by the widespread use of symptomatic medication. This needs to be further explored, as well as studies in its effectiveness for reducing cough symptoms. Meanwhile, differences in presentation and expectations to antibiotic use in the Chinese population could be further explored by comparative cross-cultural qualitative studies of patients and doctors.

### Conclusions

Clinical recovery of acute cough of around 2 weeks is usual. Overall antibiotic prescribing rates were low, but there was a high rate of antibiotic prescribing among private primary care clinicians, which warrants further exploration and scope for education and intervention. Prescription of symptomatic medications was almost universal, and further exploration on cross-cultural perceptions in the role of symptomatic and antimicrobial medication, as well as the effectiveness of symptomatic medication, is warranted.

## Materials and methods

This was a prospective multicentre observational study in Hong Kong primary care. The study protocol, case report form and patient diary were developed based on the GRACE (Genomics to combat Resistance against Antibiotics in Community-acquired LRTI in Europe, www.grace-lrti.org) project.^1^ Study documents were translated and back translated from English to Chinese, with pilot testing to ensure accuracy. The research network consisted of public primary care group clinics, private clinics and private hospital clinics, which covered New Territories, Kowloon and Hong Kong island regions of Hong Kong S.A.R Ethics review committees of the Chinese University of Hong Kong approved the study and consent forms were obtained from participants.

### Inclusion criteria

Eligible patients were aged 18 years and over who were consulting for the first time within normal consulting hours for an illness in which the main symptom was an acute or worsened cough with a duration of up to 28 days or a clinical presentation suggestive of lower respiratory tract infection (LRTI). Other requirements included being immunocompetent, the ability to fill out study materials and providing written informed consent.

### Data collection

Participating primary care physicians were asked to recruit consecutive eligible patients and to record patients’ presenting symptoms, severity and duration using a case report form. Other information collected included coexisting medical conditions (diabetes, chronic lung and cardiovascular disease), body temperature and physical examinations, medication prescription and perceived patient expectations and satisfaction and so on. The presence or absence of 14 symptoms included cough, sputum, shortness of breath, wheeze, blocked/runny nose, chest pain, fever, disturbed sleep, feeling generally unwell, muscle ache, headache, interference with normal activities/work, confusion/disorientation and diarrhoea, which were rated ‘no problem’, ‘mild problem’, ‘moderate problem’ or ‘severe problem’. Sputum production and colour were also noted.

Patients were asked to complete a daily diary for 28 days, which included daily rating of 13 symptoms until recovery, social demographic information (e.g., education, job, household size), smoking, medical history, purpose for visit, satisfaction with consultation and perceptions and expectations of antibiotic prescription.

### Sample size and patient recruitment

A conservative approach was used for sample size collection and similar to the original sample size calculation in GRACE study.^1^ Assuming a 50% probability (the most conservative estimate of probabilities in statistical terms) of antibiotic prescription, a sample size of 270 patients will have 95% confidence detecting that 50% probability. The target recruitment size was 450 to allow for a conservative estimate of 60% return rate of completed symptom diaries.

### Statistical analysis

Descriptive statistics were conducted by using means and s.d., or number (%). Symptom severities ‘none, minimal, mild, moderate and severe’ were scored 0, 1, 2, 3 and 4, respectively. Patient symptom scores of 13 symptoms were summed and scaled to range between 0 and 100 so that it could be interpreted as a percentage of maximum symptom severity. Binary logistic regression models were fitted to examine the independent effects of factors associated with antibiotic prescribing, and the influence of antibiotic use on patients’ recovery and satisfaction after controlling for age, season, smoking status, overall symptom severity, duration of illness before consultation and coexisting medical conditions. An autoregressive moving average model was fitted to predict daily symptom score of a time series from past value. Pearson's Chi-squared tests and Fisher's exact test were used for comparison in the sub-analysis of antibiotic prescriptions.

## Figures and Tables

**Table 1:** Characteristics of included patients with acute cough (*n*=321)

*Patients characteristics*	*Mean±s.d./*n *(%)*
Age (y)	47.1±14.9
Men (%)	130 (43.0%)
	
*Occupation (%)*	
Professionals/technicians/clericals	140 (43.6%)
Server/peasants/no technical workers	57 (17.8%)
Housewife/unemployed	52 (16.2%)
Retired	51 (15.9%)
Students	16 (5%)
	
*Education (%)*	
Primary school and below	45 (14.1%)
Middle school	172 (53.9%)
College and above	102 (32.0%)
Average education years (y)	11.5±4.1
	
*Smoking (%)*	
Current smoking	20 (6.3%)
Occasional smoking	8 (2.5%)
No smoking	291 (91.2%)
Sick days before this consult (d)	3.6±4.6
Self-purchased medication (%)	144 (44.9%)
Self-estimated recovery days	8.3±5.9
	
*Days of symptom recovery by diary (d)*	
Cough	9.0±6.7
Sputum	8.5±7.1
All symptoms	9.9±7.0
	
*Hospitalisation since first consult (%)*	*4 (1.32%)*
Average days of hospitalisation (d)	3.0±3.5
Re-consultation to health professional	82 (25.5%)

Abbreviations: d, days; y, years.

Data were presented as mean±s.d. for continuous variables or *n* (%) for categorical variables.

**Table 2:** Clinicians’ assessment, management and perceptions on antibiotic prescription for patients (*n*=455)

*Clinicians’ assessment, management and perceptions*	*Mean±s.d./*n*(%)*
Symptom severity scores % rated by clinicians	11.1±5.53
	
*Moderate or severe symptoms (%)*	
Cough	219 (48.1%)
Sputum	126 (27.7%)
Blocked/running nose	82 (18.0%)
Short of breath	9 (2.0%)
Wheeze	6 (1.3%)
Fever	13 (2.9%)
Muscle aching	24 (5.3%)
Headache	37 (8.1%)
Disturbed sleep	40 (8.8%)
General unwell	33 (7.3)
Patients’ oral temperature (⩾37.2 °C)	84 (18.5%)
	
*Comorbidities of patients (%)*	
COPD	16 (3.5%)
Asthma	39 (8.6%)
Other lung diseases	15 (3.3%)
Heart diseases	49 (10.7%)
Diabetes	35 (7.7%)
Antibiotic treatment (%)	31 (6.8%)
Amoxicillin	19 (4.2%)
	
*Clinicians’ perception on antibiotics (Agree or strongly agree %)*	
Patients want me to prescribe antibiotics	46 (10.2%)
Patients are satisfied with the consultation	428 (94.0%)
Antibiotics can help quick recovery	39 (8.6%)

Abbreviation: COPD, chronic obstructive pulmonary disease.

Severity scores were calculated for patients with a minimum of 85% of their symptoms recorded. The categories for clinicians to rate the severity of each symptom as ‘None’, ‘Minimal’, ‘mild’, ‘moderate’ and ‘severe’ were scored 0, 1, 2, 3 and 4, respectively. The symptom severity score was scaled to range between 0 and 100 as a percentage of maximum symptom severity. Clinicians' perceptions on antibiotics were categorised as strongly disagree, disagree, moderate, agree and strongly agree, respectively.

**Table 3:** Patients’ purpose, satisfaction and perceptions on antibiotic prescription

*Patients purpose, satisfaction and perceptions*	n *(%)*
*Patients’ main purpose of this consult*	
Concerned with illness severity	133 (41.6%)
Prescription of antibiotics	0 (0%)
Prescription of other medications	147 (45.9%)
Suggested by friends or family members	32 (10%)
Obtaining sick leave	8 (2.5%)
	
*Patients' satisfaction with the consult*	
Very unsatisfactory/ unsatisfactory	5 (4.5%)
Moderate	42 (13.4%)
Satisfactory/very satisfactory	266 (85.0%)
	
*Patients’ view on antibiotic prescription*	
Anticipation	45 (14.3%)
Expectation	32 (10.2%)
Request	9 (2.9%)
	
*Patients’ opinion on antibiotics (agree/strongly agree %)*	
I believe antibiotics are necessary for cold/cough	94 (31.0%)
I believe antibiotics have adverse effects	156 (51.3%)
I believe antibiotics use will increase resistance	199 (65.4%)

Data were presented as *n* (%). Patients’ perceptions on antibiotics were categorised as strongly disagree, disagree, moderate, agree and strongly agree, respectively.

**Table 4:** Odds ratios of factors associated with clinicians’ antibiotics prescription in patients with acute cough by logistic regression models

*Factors associated with antibiotic prescription*	*Odds ratio (95% CI)*	P *value*
*Symptom severity (Clinician’s assessment)*		
Cough	3.732 (1.944, 7.165)	<0.001
Sputum	2.699 (1.585, 4.596)	<0.001
Short of breath	2.851 (1.854, 4.384)	<0.001
Wheeze	3.229 (1.996, 5.223)	<0.001
Blocked/runny nose	0.876 (0.565, 1.360)	0.556
Fever	0.997 (0.973, 1.022)	0.837
Chest pain	0.996 (0.959, 1.035)	0.856
Muscle aching	1.562 (1.078, 2.265)	0.019
Headache	1.074 (0.730, 1.582)	0.716
Disturbed sleep	1.403 (0.985, 1.997)	0.060
Feeling generally unwell	1.848 (1.232, 2.772)	0.003
Disrupt normal activity	1.343 (0.900, 2.004)	0.149
Confusion/disoriented	6.797 (1.734, 26.649)	0.006
Diarrhoea	0.996 (0.960, 1.034)	0.853
Severity score rated by clinicians	1.113 (1.016, 1.219)	0.022
Tympanic temperature >37.2	1.854 (0.927, 3.710)	0.081
Smoking^a^	1.580 (0.796, 3.136)	0.191
		
*Clinicians’ perception on antibiotics* b		
Patients want me to prescribe antibiotics	3.263 (2.025, 5.257)	<0.001
Patients are satisfied with the consultation	3.515 (1.015, 10.231)	0.010
Antibiotics will help getting better quickly	25.946 (6.656, 101.137)	<0.001
Private primary care clinicians^c^	9.702 (2.045, 46.04)	0.004
Patient self-rated severity score at 1st day	1.007 (0.971, 1.043)	0.723
		
*Patient perceptions on antibiotics prescription* b		
Expecting antibiotics	5.106 (1.742, 11.567)	0.002
Anticipating antibiotics	5.062 (1.679, 15.023)	0.001
Requesting antibiotics	15.746 (5.019, 49.392)	<0.001
		
*Patients’ opinions on antibiotics* b		
I believe antibiotics are necessary	2.502 (1.326, 5. 163)	0.001
I believe antibiotics have adverse effects	0.762 (0.352, 1.527)	0.418
I believe antibiotics use will increase resistance	0.467 (0.287, 0.832)	0.004

Abbreviation: CI, confidence interval.

The analyses were conducted by binary logistics regression models with adjustment of age, gender, years of education, smoking, days of sickness before consult, season and comorbidities.

a Adjusted by the same covariates except smoking.

b Odds ratio further adjusted for severity scores.

c Public primary care clinicians as reference.

**Table 5:** The effect of antibiotic prescription on patients’ recovery and satisfaction

*Patients’ recovery and satisfaction*	*Antibiotics prescription*		P *value*
	*Yes*	*No*	
Patients' self-estimated overall recovery days	6.1±1.4	8.3±0.4	0.125
			
*Days of symptom recovery (mean±s.e.)*			
All symptom recovery	8.1±1.5	9.9±0.4	0.237
Cough recovery	7.1±1.5	9.1±0.4	0.195
Sputum recovery	7.3±1.5	8.5±0.4	0.445
Patient’s satisfaction (odds ratio, 95% CI)	2.766 (1.288, 3.938)	0.009	

Abbreviation: CI, confidence interval.

Data were present as mean±s.e. for adjusted means of recovery days, and odds ratio (95%CI) for patient satisfaction. The analyses were conducted by general linear models with adjustment of age, gender, season, years of education, smoking, days of sickness before consult, severity score and comorbidities. Patient satisfaction was scored 1, 2, 3, 4 and 5 for very unsatisfactory, unsatisfactory, moderate, satisfactory and very satisfactory, respectively.
