# Supplementary material for: Help-seeking and antibiotic prescribing for acute cough in a Chinese primary care population: a prospective multicentre observational study
Source: NPJ Prim Care Respir Med. 2016 Jan 21;26:15080–. doi: 10.1038/npjpcrm.2015.80 (PMC5533206; doi:10.1038/npjpcrm.2015.80)
Supplement: Supplementary Tables [file npjpcrm201580-s1.doc]

**Supplementary Table 1 Differences in antibiotic prescription by patient comorbidities** between private and public primary care clinicians

| **Patient comorbidity** |  | **Antibiotics prescription** | | **P** | **Antibiotics prescription** | | **P** |
| --- | --- | --- | --- | --- | --- | --- | --- |
|  | **Overall (n)** | **Yes** | **No** |  | **Private** | **Public** |  |
| Chronic Obstructive Pulmonary diseases | 16 | 2 | 14 | 0.360 | 1 | 1 | 0.242 |
| Asthma | 39 | 4 | 35 | 0.375 | 4 | 0 | 0.047 |
| Lung disease(other) | 17 | 2 | 15 | 0.411 | 2 | 0 | 0.007 |

**Supplementary Table 2 Differences in severity scores% between private/public clinicians and antibiotic prescription.**

|  | **Antibiotics prescription** | | | | | **Antibiotics prescription** | | | |  |
| --- | --- | --- | --- | --- | --- | --- | --- | --- | --- | --- |
| **Severity score%** | **n** | **Private** | **n** | **Public** | **P** | **n** | **Yes** | **n** | **No** | **P** |
| Clinician rated | 146 | 13.9±6.6 | 304 | 9.4±4.4 | <0.05 | 49 | 15.6±8.0 | 421 | 10.8±5.2 | <0.05 |
| Self-rated at 1^st^ day | 112 | 23.7±13.9 | 185 | 25.3±14.4 | 0.362 | 25 | 28.4±12.9 | 272 | 24.3±14.3 | 0.176 |
